# Isolation and Characterization of Extracellular Vesicles from Human and Mouse Pancreatic Tissue

**DOI:** 10.3390/bioengineering13080879

**Published:** 2026-07-30

**Authors:** Junya Peng, Lulu Liu, Xinyang Ge, Yue Han, Wenmin Tian, Yang Chen, Yupei Zhao, Xianlin Han

**Affiliations:** 1 State Key Laboratory of Complex and Severe and Rare Diseases, Peking Union Medical College Hospital, Beijing 102602, China; 2 Institute of Clinical Medicine, Peking Union Medical College Hospital, Beijing 100730, China; 3 Department of General Surgery, Peking Union Medical College Hospital, Beijing 100730, China; 4 4 + 4 Medical Doctor Program, Chinese Academy of Medical Sciences and Peking Union Medical College, Beijing 100730, China; 5 Center for Precision Medicine Multi-Omics Research, Institute of Advanced Clinical Medicine, Peking University, Beijing 100191, China; 6 Department of Biochemistry and Biophysics, Center for Precision Medicine Multi-Omics Research, School of Basic Medical Sciences, Peking University Health Science Center, Beijing 100191, China

**Keywords:** extracellular vesicles, pancreas, pancreatic cancer, tissue-derived EVs, proteomics

## Abstract

Extracellular Vesicles (EVs) have been increasingly recognized as mediators of intercellular communication in pancreatic tissues, carrying molecular cargo reflective of their cellular origin; however, their direct isolation from solid tissue remains technically challenging due to the high enzymatic activity, lipid content, and structural fragility of the pancreas. Here, we establish a pancreas-adapted and reproducible workflow for the isolation of EVs directly from human and mouse pancreatic tissues across tumor and non-tumorous contexts. This approach integrates controlled tissue processing, gentle enzymatic-mechanical dissociation, and sequential centrifugation to enable efficient vesicle recovery while preserving structural integrity and minimizing contamination. The isolated EVs exhibited characteristic size distributions, intact morphology, and enrichment of canonical EV markers, accompanied by depletion of intracellular components. Quantitative analyses demonstrated high reproducibility across independent isolations, with a coefficient of variation of less than 6%. Proteomic profiling revealed selective enrichment of vesicle-associated proteins and preservation of molecular differences between tumor and normal pancreatic tissues, indicating that tissue-derived EVs retain disease-associated protein signatures. Together, these results establish a standardized framework for pancreas-derived EV isolation that supports reproducible downstream analyses and facilitates molecular characterization of pancreatic tissues.

## 1. Introduction

Extracellular vesicles (EVs) are membrane-bound nanoscale particles released by cells and are increasingly recognized as mediators of intercellular communication [1]. By carrying proteins, lipids, and nucleic acids reflective of their cellular origin, EVs provide a valuable source of molecular information for studying tissue biology and disease processes [2]. In the context of pancreatic diseases, including pancreatic ductal adenocarcinoma (PDAC), EVs have attracted growing interest due to their potential roles in tumor progression, microenvironmental remodeling, and biomarker discovery [3,4,5].

Despite these advances, most studies of pancreatic EVs have relied on vesicle derived from cell culture systems or from disease-associated biofluids [6,7]. While these approaches are experimentally accessible, they do not fully capture the physiological complexity of intact pancreatic tissue, which consists of heterogeneous cellular populations embedded within a dense and highly structured microenvironment [8]. In particular, biofluid-derived EVs may reflect systemic signals or pathological secretomes rather than tissue-specific vesicle populations, limiting their ability to resolve localized biological processes [9].

Direct isolation of EVs from pancreatic tissue presents substantial technical challenges. The pancreas is characterized by high endogenous enzymatic activity, abundant lipid content, and structurally fragile acinar architecture, all of which can compromise vesicle integrity during tissue processing [10]. In addition, conventional tissue dissociation and isolation procedures may introduce contamination from intracellular components or lead to degradation of vesicle-associated molecules [11]. As a result, the lack of standardized and tissue-adapted isolation strategies has limited reproducibility across studies and hindered systematic molecular characterization of pancreas-derived EVs.

To address these challenges, we developed a pancreas-adapted workflow for direct EV isolation from human and mouse pancreatic tissues. The approach was systematically evaluated for EV quality, reproducibility, and compatibility with downstream proteomic analyses, providing a methodological framework for studying tissue-derived EVs in pancreatic biology and disease.

## 2. Materials and Methods

### 2.1. Ethical Approval and Human Tissue Statement

All procedures involving human tissue were conducted in accordance with the Declaration of Helsinki and approved by the Institutional Review Board (IRB) of Peking Union Medical College Hospital (Approval No. I-26PJ0272).

### 2.2. Reagents and Equipments

The main reagents, consumables, antibodies, and instruments used for pancreatic tissue-derived EV isolation and characterization are summarized in Table 1.

### 2.3. Buffer

Preservation Solution 1×, 100 mL: RPMI 1640 medium with 1 mM protease inhibitor cocktail.

Digestion Solution 1×, 50 mL: phosphate-buffered saline (PBS) containing 5% (*v*/*v*) EV-depleted fetal bovine serum (FBS; prepared by ultracentrifugation at 120,000× *g* for 18 h), 1 mg/mL Type VIII collagenase, 2 mg/mL Dispase II, 1 mg/mL trypsin inhibitor, and 1 unit/mL DNase I, pH adjusted to 7.4 if necessary.

Wash Buffer 1×, 500 mL: phosphate-buffered saline (PBS) with 0.04% (*w*/*v*) bovine serum albumin (BSA).

### 2.4. Tissue Collection and Preparation

Human pancreatic ductal adenocarcinoma (PDAC) tissues and paired histologically normal pancreatic tissues were obtained from the same patients undergoing surgical resection at Peking Union Medical College Hospital. Matched normal tissues were dissected from non-tumorous regions at least 2 cm away from the tumor margin, whereas PDAC tissues were harvested from non-necrotic areas of primary tumor lesions. Fresh tissue specimens (150–300 mg or 1–2 cm^2^ per sample) were collected immediately after resection, anonymized, and transported on ice in sterile ice-cold Preservation Solution to prevent tissue autolysis. Within 20 min of collection, adipose and necrotic tissues were removed; all samples were rinsed three times with cold PBS and transferred to Digestion Solution, then minced into 1–2 mm^3^ fragments for immediate processing.

Murine pancreatic tissues were isolated from 6-week-old female C57BL/6 mice in accordance with institutional animal care and use committee guidelines (XHDW-2025-222). Following euthanasia, the pancreas was excised without in situ perfusion and immediately immersed in cold Preservation Solution at concentrations identical to those used for human tissues. Tissues were washed thoroughly, cleared of visible fat and blood clots, and transferred to Digestion Solution, then minced on ice into small fragments of 1–2 mm^3^.

For EV characterization, three biological replicates of human normal pancreatic tissues, three human PDAC tissues, and three normal mouse pancreatic tissues were analyzed by nanoparticle tracking analysis (NTA), transmission electron microscopy (TEM), and zeta potential measurement. Western blot analysis was performed using three normal mouse pancreatic tissues samples and four human PDAC tissue samples.

All procedures were performed on ice to minimize proteolytic degradation and preserve the structural integrity of EVs before isolation.

### 2.5. Tissue Dissociation

Pancreatic tissue fragments (1–2 mm^3^) were transferred to 60 mm × 20 mm culture dishes and immersed in approximately 5 mL of digestion buffer per dish. Dishes were incubated at 37 °C for 5 min without agitation. After incubation, the mixture was gently triturated approximately 20 times using wide-bore pipette tips to facilitate the release of cells and EVs. The dissociation process was monitored by bright-field microscopy after each trituration cycle to ensure adequate tissue fragmentation while avoiding excessive mechanical disruption. A detailed optimization of this trituration procedure has been reported previously [12]. This incubation–trituration cycle was repeated until a homogeneous, low-viscosity cell-EV suspension was obtained. Typically, 2–4 cycles were sufficient for tissue dissociation. Care was taken to avoid over-digestion, with total enzymatic exposure restricted to ≤20 min for murine tissues and human tissues.

After each cycle, the suspension was transferred to a 50 mL conical tube and allowed to settle for approximately 30 s to sediment large tissue fragments, and the supernatant was collected. Supernatants from consecutive cycles were combined and adjusted to a desired volume using PBS supplemented with 5% EV-depleted FBS. Following tissue dissociation by pipette trituration, the resulting suspension was passed through a 100 μm cell strainer using a wide-bore pipette tip to remove residual tissue debris and large particulates. Contaminating red blood cells were lysed by incubation with pre-chilled RBC lysis buffer on ice for 5 min, followed by neutralization with excess ice-cold PBS.

### 2.6. Sequential Pre-Clearance

All centrifugation steps were performed at 4 °C. The filtered suspension was subjected to the following sequential spins:

Step1: 300× *g* for 20 min—to pellet intact cells. The supernatant was carefully transferred to a new pre-chilled tube, leaving the cell pellet behind. The resulting pellet was collected, washed with wash buffer and centrifuged at 600× *g* for 5 min twice, and lysed with RIPA buffer supplemented with protease inhibitors to generate whole-cell lysates (WCL) for western blotting and proteomic analyses.

Step2: 3000× *g* for 20 min—to pellet coarse debris (e.g., large membrane fragments, nuclei). The supernatant was transferred to a fresh tube.

Step3: 10,000× *g* for 40 min—to pellet apoptotic bodies, large EVs, and other subcellular particles. The resulting supernatant constituted the pre-cleared EV-containing fraction.

After final centrifugation, the pellet was collected and washed by wash buffer twice, then RIPA buffer supplemented with PMSF was added.

### 2.7. EV Isolation and Purification

After the preceding tissue processing and differential centrifugation steps, the resulting supernatants were collected and used for EV isolation. Before ultracentrifugation, the supernatants were passed through 0.22 μm pore-size PES filters (Millipore). The filtered solutions were first ultracentrifuged at 110,000× *g* for 70 min at 4 °C using a Beckman 45 Ti rotor. The resulting pellets were then gently resuspended in 1 mL PBS and subjected to a second ultracentrifugation step at 110,000× *g* for 70 min at 4 °C using a Beckman TLA-55 rotor to wash the pellet once. After discarding the supernatant, the washed pellet was finally resuspended in 30 μL PBS, yielding the purified pancreatic EVs.

### 2.8. EV Characterization

Purified extracellular vesicles (EVs) were characterized following the MISEV2023 guidelines to confirm particle size, morphology, and protein marker expression [13].

#### 2.8.1. Transmission Electron Microscopy (TEM)

The pancreatic EV pellet was resuspended in 30 μL of PBS. Electron microscopy grade glutaraldehyde was added to the suspension to a final concentration of 2.5% for fixation. After fixation, glow-discharged copper grids were floated on the sample droplet. One minute later, the residual liquid was removed with lint-free paper, and the same grid was negatively stained with 1% uranyl acetate for 1 min, after which the stain was removed. All grids were examined using a Hitachi HT7800 transmission electron microscope (Hitachi High-Technologies Corporation, Tokyo, Japan).

#### 2.8.2. Nanoparticle Tracking Analysis (NTA)

NTA was carried out using a ZetaView system (Particle Metrix, Meerbusch, Germany). Samples were diluted in advance to reach an optimal concentration of 100–200 particles per frame. For each EV sample, measurements were performed in triplicate across 11 distinct positions within the analysis cell under the following settings: temperature maintained at 25 °C, sensitivity at 80, shutter at 100, maximum area limit of 1000, minimum area limit of 10, and minimum brightness set to 25. A minimum of 1000 valid particle tracks was acquired per run to ensure reliable results. Data acquisition and analysis were performed with Software ZetaView (version 8.05.16 SP3).

#### 2.8.3. Zeta Potential (ζ) Measurement

Zeta potential was measured using a Zetasizer Pro system (Malvern Panalytical, Malvern, UK). Purified pancreatic EVs were resuspended in PBS to a final protein concentration of 1 mg/mL. An appropriate aliquot of the sample was loaded into a disposable folded capillary cell, with care taken to avoid air bubbles. All measurements were performed at a controlled temperature of 25 °C following an equilibration time of 120 s. The voltage and measurement duration were automatically optimized by the ZS Xplorer software (version 3.1.0.64), and the constant current mode was enabled. Each independent sample was measured in triplicate.

#### 2.8.4. Immunoblotting Analysis

EV lysates were prepared using RIPA buffer supplemented with protease inhibitors. Equal amounts of total protein (20 μg) were separated by SDS-PAGE (10–12%), transferred to PVDF membranes, and incubated with primary antibodies against TSG101, Calnexin, and β-actin at 4 °C for 16 h. After washing with TBS-T, the membranes were incubated with HRP-conjugated secondary antibodies for 1 h at room temperature, washed again with TBS-T, and finally visualized using an enhanced chemiluminescence (ECL) kit.

### 2.9. Data Analysis and Quality Control

Data from NTA, Western blotting, and TEM were analyzed to ensure the reproducibility and purity of extracellular vesicle (EV) preparations.

Particle quantification and normalization. Particle concentration and size distribution obtained from NTA were normalized to the initial tissue input (expressed as total particles per 100 mg of tissue). The mean and standard deviation were calculated from at least three independent isolations. Reproducibility of particle yield and size was assessed using the coefficient of variation (CV), which was required to remain below 15%.

Protein-to-particle ratio. Total protein concentration of each EV preparation was measured using the BCA assay and compared to particle number determined by NTA. A low protein-to-particle ratio was considered indicative of high-purity EV preparations. Samples with excessive soluble protein content relative to vesicle count were excluded from downstream analyses.

Western blot validation. Each EV preparation was verified for the presence of canonical EV markers and the absence of non-vesicular contaminants. Only preparations meeting these criteria were accepted as qualified EV samples.

Batch reproducibility. At least three independent isolations were performed for each tissue type (human and mouse). EV preparations were considered reproducible when NTA profiles, TEM morphology, and marker expression patterns remained consistent across batches within the defined variation limits.

All quantitative analyses were conducted using standardized acquisition and processing parameters, and data evaluation was performed in a blinded manner to minimize operator bias. These procedures ensured that EV preparations were consistent, reproducible, and compliant with MISEV2023 guidelines for extracellular vesicle quality assessment.

### 2.10. DIA-MS-Based Proteomic Profiling of Pancreas-Derived EVs

EV pellets (20–40 µg protein per sample) were lysed in 8 M urea with 100 mM Tris-HCl (pH 8.0) containing protease inhibitors, followed by reduction with 10 mM dithiothreitol (DTT, 37 °C for 1 h) and alkylation with 20 mM iodoacetamide (IAA, room temperature for 30 min in the dark). Samples were diluted fourfold with 50 mM ammonium bicarbonate and digested overnight with sequencing-grade trypsin at an enzyme-to-protein ratio of 1:50.

Peptides were desalted using C18 StageTips, dried under vacuum, and reconstituted in 0.1% formic acid for LC–MS analysis. Peptide separation was performed on an Easy-nLC 1200 system coupled to an Orbitrap Q Exactive HF-X mass spectrometer. All samples were separated within 120 min on a reversed-phase C18 column (25 cm × 75 μm, 1.7 μm, IonOpticks). The elute gradient of B was linearly increased from 5% to 30% within 109 min, followed by an increase to 95% within 5 min and continued for another 6 min at a flow rate of 300 nL/min at 50 °C (Mobile phases A: 0.1% formic acid in water; Mobile phases B: 0.1% formic acid in 80% acetonitrile). For single-shot proteomics with data-independent acquisition (DIA), the MS1 scan resolution was set to 60,000. The MS1 scan range was 350–1200 *m*/*z*, the AGC target was 3 × 10^6^, and the maximum injection time was 50 ms. Precursors were isolated with a dynamic isolation width covering 350–1200 *m*/*z*. Precursors were fragmented precursors at an NCE of 28. MS2 scans were acquired by Orbitrap at resolution of 30,000. The maximum injection time was set to auto, AGC target was set to 1 × 10^6^.

Spectral libraries were generated with Spectronaut version 18.0 (Biognosys) against the corresponding reviewed UNIPROT human and mouse database. DIA files were processed in a default mode (Tables S1 and S4). Protein identifications were filtered to a 1% false discovery rate (FDR) at both peptide and protein levels. Label-free quantification was performed using intensity-based absolute quantification (iBAQ) values. Protein abundance matrices exported from Spectronaut were normalized using OmicsBean (Tables S2 and S5). All downstream analyses, including differential protein abundance analysis between EV and WCL fractions, and gene ontology enrichment, were performed in R and GraphPad Prism 10. Differentially enriched proteins were defined using |log_2_ fold change| > 1 and *p* < 0.05 (Tables S3 and S6). EV-enriched and WCL-enriched protein lists were separately subjected to Gene Ontology biological process enrichment analysis using DAVID. Enrichment results and representative pathway-associated proteins were visualized using R.

## 3. Results

### 3.1. Workflow Overview and Study Design Across Tissue Types and Species

**Figure 1:**
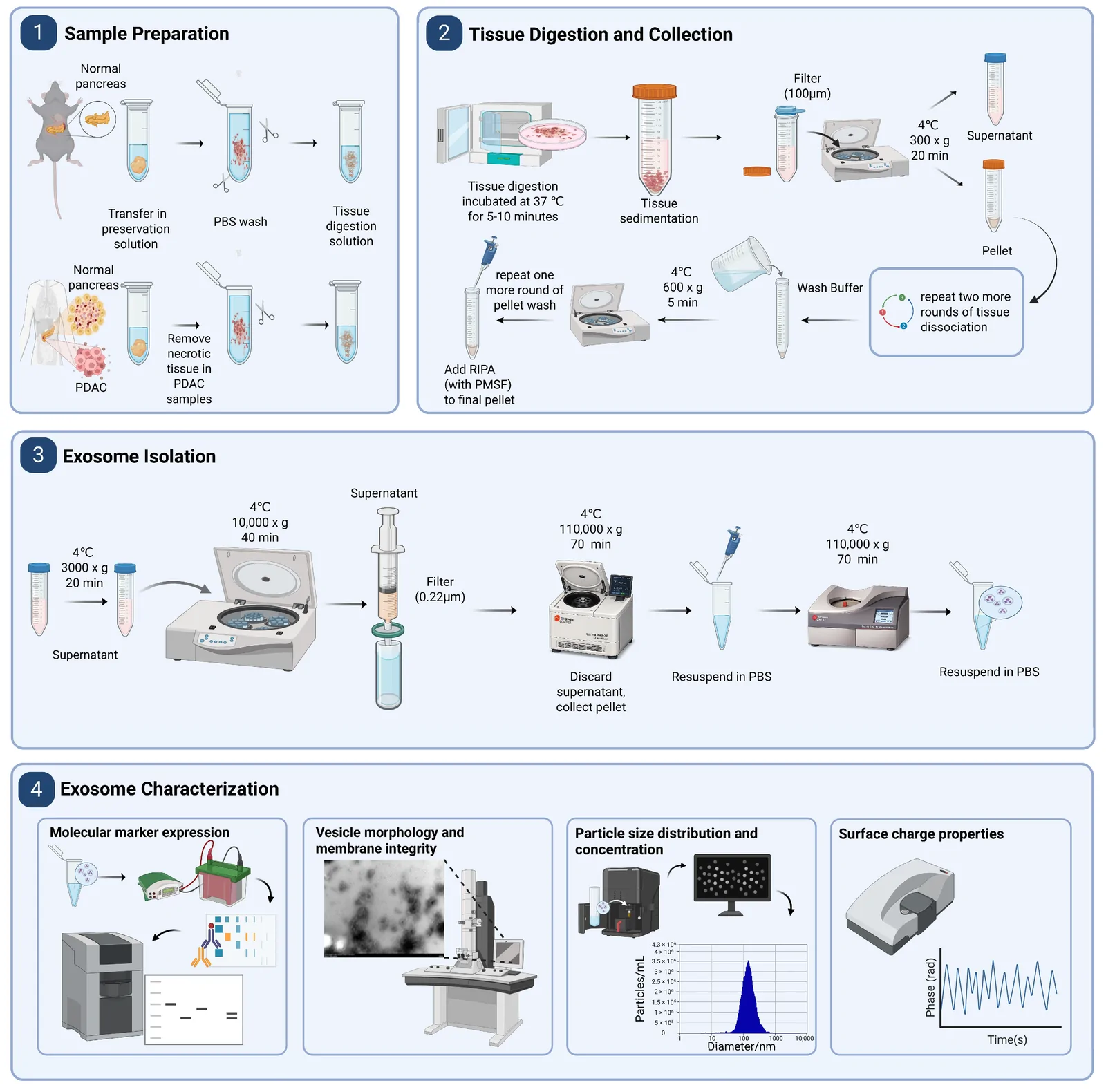
Schematic illustration of the four-step workflow showing sample preparation, tissue digestion and collection, exosome isolation, and extracellular vesicles characterization.

A schematic summary of the isolation and characterization workflow is presented in Figure 1. Fresh pancreatic tissues were rapidly collected and processed under cold, protease-inhibiting conditions to minimize ischemic damage and preserve vesicle integrity. Samples were trimmed to remove adipose and necrotic regions, ensuring that only viable tissue was subjected to downstream processing. Vesicles were released through gentle enzymatic-mechanical dissociation, followed by sequential low-speed centrifugation steps to remove intact cells, apoptotic bodies, and residual debris, yielding a clarified supernatant enriched in small EVs. Subsequent ultracentrifugation enabled the recovery and concentration of nanosized vesicles. The isolated vesicles were then characterized by western blotting, transmission electron microscopy, and NTA to assess EV-specific protein marker expression, vesicle morphology and membrane integrity, and particle size distribution and concentration, respectively, in accordance with MISEV2023 guidelines [13]. Zeta potential measurement is included as an optional parameter to evaluate surface charge and colloidal stability.

To evaluate the robustness and general applicability of the workflow, EV isolation was performed across multiple biological contexts, including human pancreatic tumor tissues, matched adjacent non-tumorous tissues, and normal mouse pancreas. This design enabled systematic assessment of the workflow across distinct tissue microenvironments and across species. The entire workflow—from tissue acquisition to EV validation—can be completed within approximately 30 h for a batch of up to 6 samples, providing a standardized, reproducible, and moderate-throughput approach for isolating high-quality EVs from pancreatic tissues.

### 3.2. Quality Validation and Reproducibility of Pancreas-Derived EVs

To comprehensively evaluate the quality of the isolated extracellular vesicles (EVs), we performed complementary analyses assessing molecular marker expression, vesicle morphology and membrane integrity, particle size distribution and concentration, and surface charge properties. These analyses were performed across different pancreatic tissue sources, including human PDAC tissues, matched adjacent non-tumorous pancreatic tissues, and normal mouse pancreas, to evaluate both EV quality and workflow reproducibility. These measurements collectively validated vesicle identity, structural integrity, purity, and colloidal stability.

**Figure 2:**
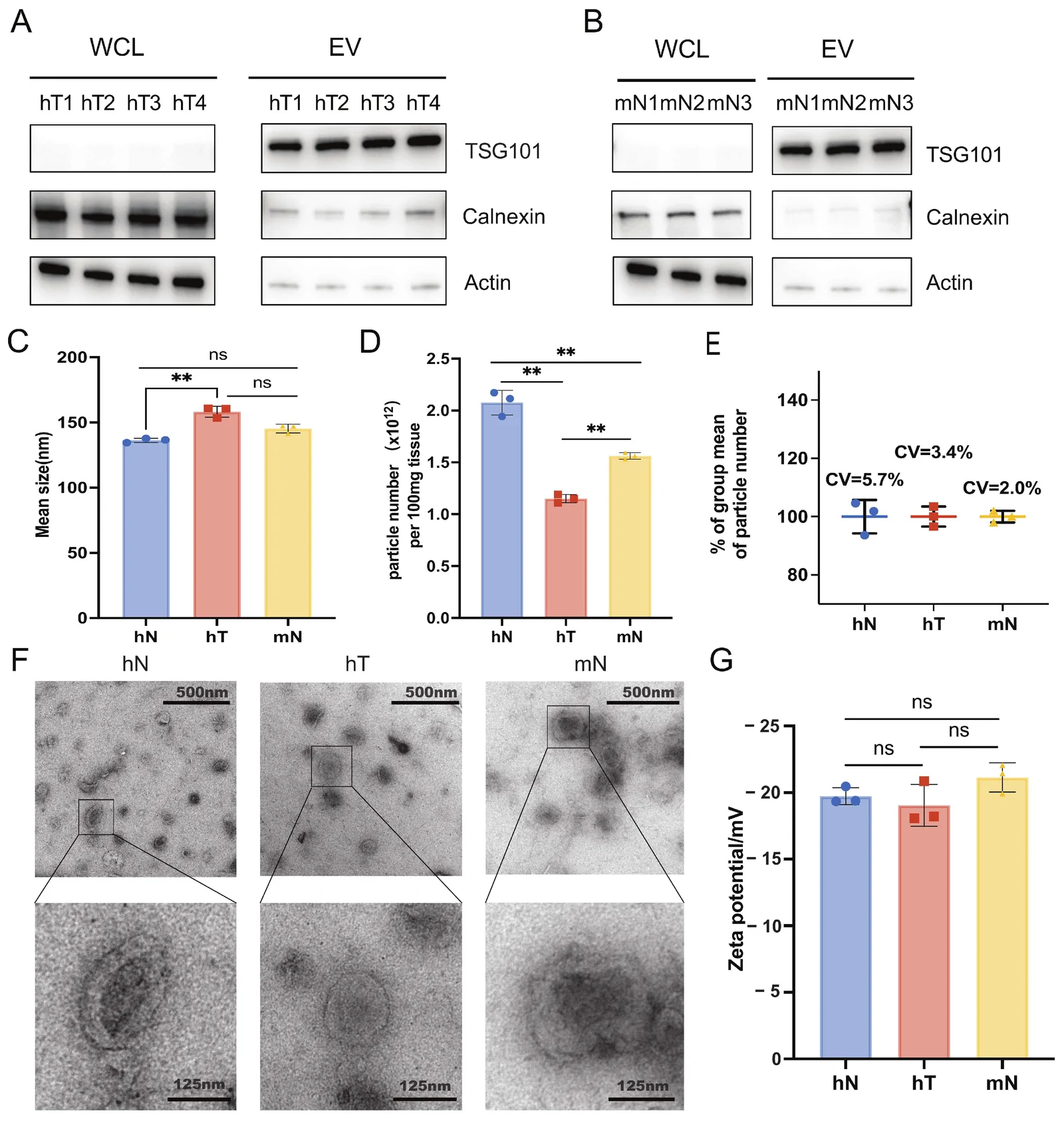
Characterization of extracellular vesicles isolated from human pancreatic tissues and mouse pancreatic tissues. (**A**,**B**) Western blot analysis of EV marker proteins (TSG101) and negative contaminant markers (Calnexin, endoplasmic reticulum; β-Actin) in whole-cell lysates (WCL) and purified EVs from human PDAC tissues (hT1-hT4) and mouse-derived pancreas samples (mN1-mN3). (**C**) Nanoparticle tracking analysis (NTA)-determined average particle diameter of EVs isolated from human samples (hN, hT)and mouse samples (mN) groups; hT-derived EVs exhibited significantly larger mean size versus hN (** *p* < 0.01), no significant difference (ns) was found between hT and mN groups. (**D**) Quantification of total EV particle yield normalized to per 100 mg source tissue. EV abundance was markedly decreased in hT group compared with hN (** *p* < 0.01). (**E**) Coefficient of variation (CV) statistics of EV particle number across biological replicates in three groups, with CV values of 5.7% (hN), 3.4% (hT) and 2.0% (mN), indicating good experimental repeatability of EV isolation. (**F**) Transmission electron microscopy (TEM) micrographs of purified EVs from hN, hT and mN groups; magnified insets display typical cup-shaped bilayer membrane morphology characteristic of small extracellular vesicles, scale bars: upper = 500 nm, lower = 125 nm. (**G**) Zeta potential detection of isolated EVs; all three groups presented uniformly negative surface potential with no statistically significant intergroup differences (ns). Data are presented as mean ± SD; **, *p* < 0.01; ns, no significant difference.

Western blot analysis was performed to validate EV-specific protein enrichment and sample purity by comparing EV fractions with corresponding whole-cell lysates derived from human PDAC tumor tissues and normal mouse pancreas. EV preparation exhibited enrichment of the EV-associated protein TSG101 relative to whole-cell lysates (Figure 2A,B). In contrast, the endoplasmic reticulum marker Calnexin was markedly depleted in EV fractions, indicating minimal contamination from intracellular compartments. Actin, used as a cellular protein reference, was also reduced in EV preparations compared with whole-cell lysates. Consistent enrichment of EV-associated markers and depletion of non-vesicular proteins across independent preparations further supported the reproducibility of the isolation workflow.

NTA was used to determine particle size distribution and concentration of EVs isolated from human PDAC tumor tissues, matched adjacent non-tumorous tissues, and normal mouse pancreas. Across all sample types, EV preparations exhibited a unimodal size distribution with a modal diameter of 134.6 nm (mean: 146.6 ± 9.6 nm), predominantly within the range of 77.7–223.3 nm, consistent with the expected size profile of small extracellular vesicles (Figure 2C). Particle concentrations normalized to tissue input were within the expected range for tissue-derived EV preparations [14], with values of 2.1 × 10^12^, 1.2 × 10^12^, and 1.6 × 10^12^ particles per 100 mg tissue for adjacent, tumor, and mouse pancreas samples, respectively (Figure 2D). The recovery yield showed low variability among independent isolation (CV values ranging from 2.0% to 5.7%; Figure 2E), indicating stable EV recovery across different tissue contexts. Variation among tissue sources likely reflects biological differences rather than technical inconsistency. Together, these results indicate that the workflow enables reliable recovery of EVs with characteristic size distribution and particle yield across diverse pancreatic tissue sources.

Transmission electron microscopy (TEM) was applied to examine vesicle morphology and membrane integrity. TEM imaging revealed a homogeneous population of vesicle with typical cup-shaped morphology, continuous lipid bilayers, and smooth surface contours (Figure 2F). No evidence of aggregation, membrane disruption, or cellular debris was observed, indicating preservation of vesicle ultrastructure during the isolation process. The consistent preservation of vesicle morphology across different tissue sources further demonstrated the robustness of the isolation procedure. These observations are consistent with the expected structural characteristics of small extracellular vesicles.

Zeta potential analysis was performed as an auxiliary physicochemical assessment to evaluate the surface charge and colloidal stability of EVs isolated from human PDAC tumor tissues, matched adjacent non-tumorous tissues, and normal mouse pancreas. The measured zeta potential values were consistently negative (−20.0 ± 1.4 mV across all sample types) (Figure 2G), in line with the expected surface properties of lipid bilayer-enclosed vesicles [15,16]. No substantial variation in zeta potential was observed among the three tissue sources, suggesting stable and non-aggregating EV preparations.

Collectively, these complementary analyses demonstrate that the established workflow consistently generates pancreatic tissue-derived EV preparations with comparable molecular characteristics, morphology, particle properties, and physicochemical stability across isolation batches, tissue types, and species, supporting its reproducibility and applicability for downstream comparative analyses.

### 3.3. Downstream Compatibility: Proteomic Profiling of Pancreas-Derived EVs

**Figure 3:**
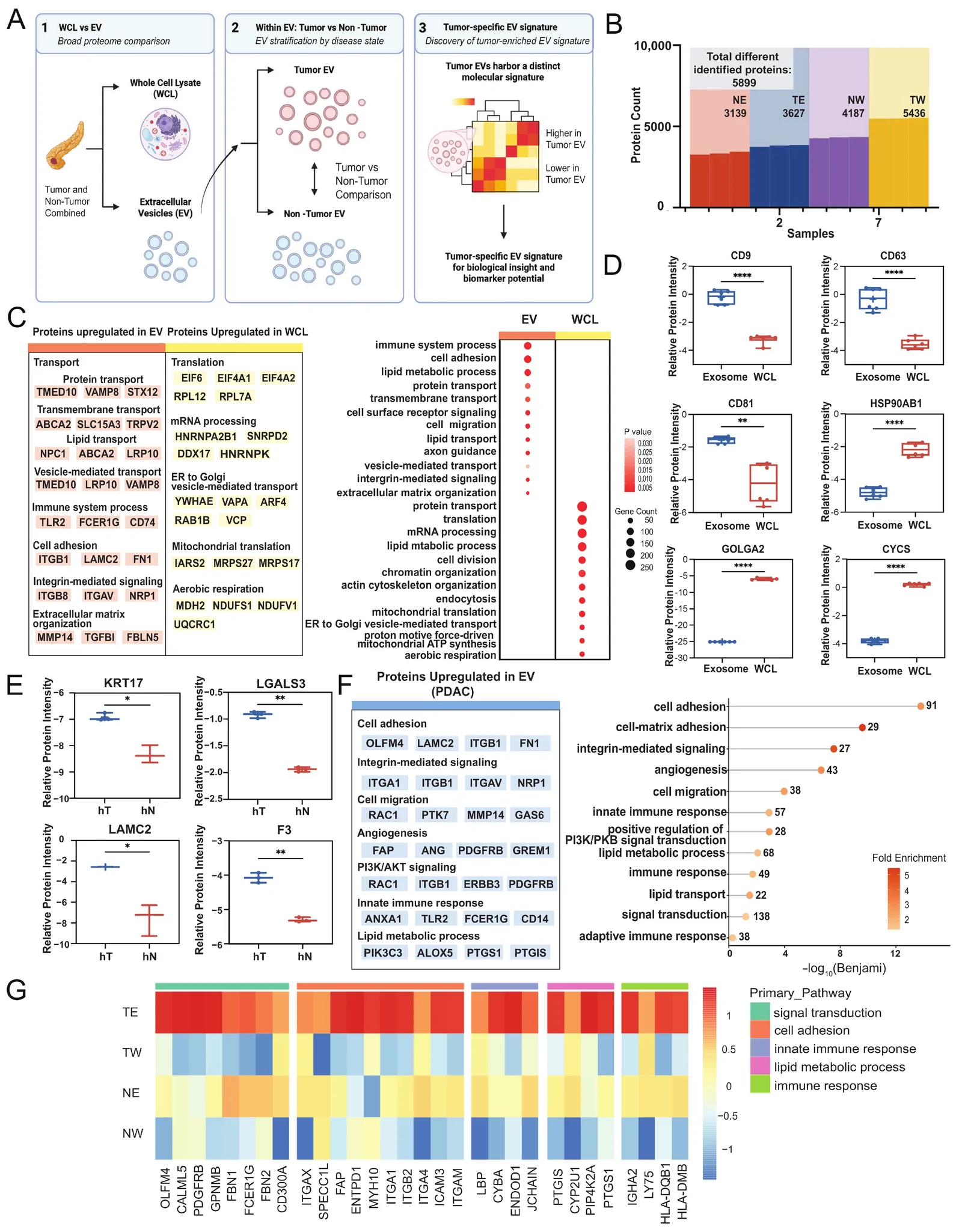
Proteomic characterization of human pancreatic tissue-derived EV and WCL fractions. (**A**) Schematic overview of the experimental design and analytical workflow. WCL and EVs were isolated from paired human pancreatic tumor and adjacent non-tumor tissues, followed by broad proteome comparison, EV-focused stratification, and identification of tumor-associated EV signatures. (**B**) Summary of the total number of differentially identified proteins across human sample groups, including tumor WCL (TW), tumor EVs (TE), non-tumor WCL (NW), and non-tumor EVs (NE). (**C**) Functional classification of proteins upregulated in human EV and WCL fractions. Representative enriched pathways and associated proteins are shown on the left, and Gene Ontology biological process enrichment is summarized as a bubble plot on the right. Dot size indicates gene count, and color represents enrichment *p* value. (**D**) Relative protein intensity of canonical EV-associated proteins (CD9, CD63, CD81) and intracellular contaminant markers (HSP90AB1, GOLGA2, CYCS) in human EV and WCL fractions. (**E**) Relative protein intensity of selected tumor-associated EV proteins (KRT17, LGALS3, LAMC2, F3) comparing human tumor-derived EVs and human non-tumor EVs. (**F**) Functional annotation of proteins upregulated in human PDAC-derived EVs. Representative proteins were grouped according to major biological processes. The bubble plot summarizes enriched biological pathways (including cell adhesion, cell-matrix adhesion, integrin-mediated signaling, angiogenesis, cell migration, innate immune responses, PI3K/AKT signaling, and lipid metabolic processes), with dot size indicating gene count and color indicating fold enrichment. (**G**) Heatmap showing representative pathway-associated proteins across human tumor EVs (TE), human tumor WCL (TW), human non-tumor EVs (NE), and human non-tumor WCL (NW). Statistical significance was determined by a paired two-tailed Student’s *t*-test unless otherwise indicated. * *p* < 0.05, ** *p* < 0.01, **** *p* < 0.0001.

To evaluate the downstream compatibility of the isolated EVs for proteomic analysis, we performed mass spectrometry-based profiling across a structured sample set designed to assess both enrichment specificity and biological signal preservation, including human pancreatic tumor and normal tissues with matched whole-cell lysates and EV fractions, as well as mouse normal pancreatic tissue (Figure 3A).

Across all samples, a total of 5899 proteins were identified, demonstrating robust proteome coverage across both whole-cell lysates and EV fractions (Figure 3B). Whole-cell lysates yielded approximately 4000–5500 proteins, while EV fractions consistently yielded ~3200–3900 proteins across human and mouse samples, reflecting the expected reduction in proteome complexity associated with vesicle-associated protein subsets. Importantly, protein identification was highly consistent across biological replicates within each sample group, supporting the robustness and stability of the dataset. Protein detection remained stable across species (human and mouse) and tissue conditions (tumor and normal), indicating consistent proteome depth under the established workflow.

To further characterize the protein composition of EV fractions, we performed enrichment analysis comparing EVs and whole-cell lysates derived from human pancreatic tissues, with tumor and normal samples analyzed jointly to capture overall EV-associated proteomic features. Gene Ontology analysis revealed that EV fractions were significantly enriched for proteins associated with extracellular vesicles, membrane components, and vesicle-mediated transport, while proteins related to intracellular compartments were comparatively depleted (Figure 3C). Consistent with this global enrichment pattern, examination of individual protein abundance further confirmed the selective enrichment of canonical EV-associated proteins, including CD9, CD63, and CD81 [11,17], within EV fractions (Figure 3D). In contrast, intracellular proteins associated with cytosol, Golgi apparatus and mitochondria, including HSP90AB1, GOLGA2, and CYCS [18], were reduced in EV fractions relative to whole-cell lysates. A comparable enrichment pattern was observed when comparing EV fractions and whole-cell lysates in mouse pancreatic samples, further supporting the consistency of EV isolation-associated proteome selection across species (Figure S1). Together, these results demonstrate that the isolated EVs exhibit a characteristic vesicle-associated proteomic profile, supporting effective enrichment of membrane-bound vesicles with minimal contamination from bulk cellular proteins.

To further evaluate whether the isolated EVs preserve biologically relevant molecular features, we compared EV proteomes derived from human pancreatic tumors and matched normal tissues. At the level of established markers, tumor-derived EVs exhibited increased abundance of proteins associated with pancreatic epithelial malignancy, including KRT17, LAMC2, LGALS3, and F3, relative to normal pancreatic EVs (Figure 3E). These findings are consistent with known molecular features of pancreatic tumor cells [19,20,21,22], supporting the biological fidelity of the isolated EVs.

Beyond these established markers, comparative proteomic analysis further revealed additional proteins differentially represented between tumor- and normal-derived EVs. At the global level, enrichment analysis highlighted functional categories associated with cell adhesion, integrin-mediated signaling, angiogenesis, cell migration, innate immune responses, PI3K/AKT signaling, and lipid metabolic processes, indicating that EV-associated proteomes capture molecular programs related to tumor progression and microenvironmental remodeling (Figure 3F). At the level of individual proteins, several coordinated protein modules were enriched in tumor-derived EVs, including OLFM4 [23,24], FAP [25], JCHAIN, PTGIS, and HLA-DQB1, exhibited differential representation in tumor-derived EVs compared to normal pancreatic EVs, reflecting broader molecular features associated with the disease state (Figure 3G).

Together, these results demonstrate that the isolated EVs are compatible with downstream proteomic analysis, retaining both biochemical specificity and biologically meaningful protein signatures. This supports their utility as a reliable platform for molecular characterization and biomarker discovery in pancreatic cancer.

## 4. Discussion

In this study, we established a standardized and reproducible workflow for isolating extracellular vesicles from pancreatic tissues across species and biological contexts. By integrating controlled tissue processing, stepwise dissociation, and sequential centrifugation, the approach enables efficient recovery of EVs from complex solid tissue environments while preserving vesicle integrity and minimizing contamination. Comprehensive validation across multiple orthogonal measurements—including molecular markers, particle size distribution, ultrastructural morphology, and physicochemical properties—demonstrated that the isolated vesicles meet key criteria for bona fide EVs. Importantly, the consistency observed across independent isolations, tissue types, and species highlights the robustness and general applicability of the workflow. Beyond technical validation, our results further demonstrate that the isolated EVs are compatible with downstream molecular analyses. Proteomic profiling not only confirmed effective enrichment of vesicle-associated proteins but also revealed preservation of biologically relevant differences between tumor and normal tissues, indicating that tissue-derived EVs can serve as an informative substrate for comparative and discovery-oriented studies.

Existing approaches for pancreatic extracellular vesicle isolation can be broadly classified into three categories, each with inherent limitations. First, protocols based on cell line-derived conditioned medium, typically using pancreatic cancer or immortalized ductal cell lines, are technically straightforward and yield relatively homogeneous vesicle populations, but fail to recapitulate the physiological complexity and multicellular interactions present in intact pancreatic tissue [26]. Second, EV isolation from pancreatic disease-associated fluids, such as ascites or cystic fluid, has been reported [27]. While these approaches are technically feasible, they reflect pathological secretomes rather than the physiological vesicle milieu of normal pancreatic tissue. Third, generic solid tissue-based protocols have been applied across multiple organ systems [28,29,30]; however, they are not specifically optimized for pancreatic tissue, which is characterized by high endogenous enzymatic activity, abundant lipid content, and a structurally fragile acinar architecture. As a result, these approaches may compromise vesicle integrity, promote degradation of surface proteins, and increase co-isolation of non-vesicular components. Collectively, these limitations highlight the lack of a standardized and tissue-adapted strategy for isolating extracellular vesicles directly from pancreatic tissue under physiological conditions, which has contributed to variability across studies and limited the systematic characterization of pancreas-derived EVs.

To overcome these limitations, we developed a pancreas-specific workflow with demonstrated cross-species applicability. The protocol was optimized to accommodate the biochemical and structural complexity of the pancreas, an organ rich in digestive enzymes, lipids, and fragile acinar structures that challenge vesicle preservation. By integrating gentle enzymatic-mechanical dissociation with targeted protease inhibition under cold and time-controlled conditions, the workflow minimizes membrane disruption and enzymatic degradation while maintaining physiological vesicle release. Importantly, this pancreas-adapted design enables efficient recovery of intact vesicles from both tumor and non-tumorous pancreatic tissues, capturing EV populations derived from diverse cellular compartments within the tissue microenvironment. It should be noted that tissue-derived EV isolation approaches may recover EV populations originating from multiple cellular sources within the tissue microenvironment. Although the current workflow minimizes contamination from intact cells and tissue debris, the precise contribution of EVs from different cellular compartments, including potential residual circulating EVs, cannot be completely resolved. Therefore, the isolated vesicles are interpreted as tissue-derived EV populations associated with the pancreatic microenvironment rather than a single defined EV subtype. This reduces the risk of cellular debris contamination commonly associated with generic tissue protocols. Moreover, the method performed robustly in both human and mouse pancreas, yielding comparable vesicle morphology, size distribution, and marker expression. Such cross-species reproducibility, together with consistent performance across tumor and normal tissues, not only validates the robustness of the procedure but also provides a unified methodological platform for translational comparison between preclinical models and human pancreatic disease.

In addition to its tissue-adapted design, another key strength of this workflow lies in its ability to preserve biologically meaningful EV-associated molecular features. Unlike many isolation strategies in which extensive processing may alter vesicle composition or introduce bias, the present approach maintains EV cargo integrity, enabling the retention of physiologically relevant protein signatures. This is evidenced by the consistent enrichment of vesicle-associated proteins and the preservation of biologically relevant differences between tumor and normal pancreatic tissues observed in downstream proteomic analyses. These findings suggest that the isolated EVs not only reflect their cellular origin but also capture disease-associated molecular variation within the tissue microenvironment. As a result, this workflow provides a reliable platform not only for EV isolation but also for downstream comparative and discovery-oriented analyses, particularly in the context of pancreatic cancer.

Beyond its technical advantages, our findings provide insight into the biological relevance of tissue-derived EVs in pancreatic systems. The ability of EV preparations to retain distinct proteomic differences between tumor and non-tumorous tissues suggests that vesicle cargo reflects the cellular and microenvironmental composition of the pancreas. Given the complex interplay between epithelial, stromal, and immune compartments in pancreatic tissue, EVs may serve as integrative carriers of molecular information across these cellular contexts. Importantly, the preservation of disease-associated molecular features in tumor-derived EVs supports the concept that EVs isolated directly from tissue can provide a more localized and physiologically relevant snapshot of cellular states compared to circulating vesicles. This highlights the potential of tissue-derived EVs as a platform for investigating tumor biology and microenvironmental interactions in pancreatic disease.

From a translational perspective, the ability to isolate high-quality EVs directly from pancreatic tissue enables systematic exploration of EV-associated molecular signatures in disease contexts. The preservation of both established and data-driven proteomic features suggests that tissue-derived EVs may represent a valuable source of candidate biomarkers for pancreatic cancer detection and stratification. Furthermore, the demonstrated compatibility of this workflow across human and mouse tissues provides a unified framework for linking preclinical models with human disease, facilitating cross-species validation of EV-associated markers. Such a platform may support future efforts to translate EV-based discoveries into clinically relevant diagnostic and prognostic applications.

Several limitations of this study should be acknowledged. First, the sample size for proteomic analysis remains limited, and further studies in larger and clinically diverse cohorts will be required to validate the generalizability of EV-associated molecular features. Second, while the present work demonstrates preservation of EV cargo at the molecular level, functional characterization of these vesicles was not addressed, and will be necessary to determine their biological roles in pancreatic physiology and disease. In addition, although the workflow minimizes contamination and preserves vesicle integrity, it does not explicitly resolve distinct EV subpopulations, and future studies incorporating higher-resolution isolation or single-vesicle approaches may further refine the characterization of pancreas-derived EVs.

## Figures and Tables

**Table 1:** Reagents and equipments.

Category	Reagent/Material	Specification/Supplier
Buffers and Basic Solutions	Phosphate-buffered saline (PBS, pH 7.4)	Hyclone, Cytiva, Logan, UT, USA, Cat. No. SH30256.01
	RPMI 1640 Medium	Gibco, Thermo Fisher Scientific, Grand Island, NY, USA, Cat. No. 11875-093
	Fetal bovine serum (FBS)	Gibco, Cat. No. 16000-044
Tissue Dissociation Enzymes	Protease inhibitor cocktail	Solarbio, Beijing, China, Cat. No. P6730
	Bovine serum albumin (BSA)	Sigma-Aldrich, St. Louis, MO, USA, Cat. No. B2064
	RBC lysis buffer	Invitrogen, Thermo Fisher Scientific, Waltham, MA, USA, Cat. No. 1966634
	DNase/RNase-free water	Molecular biology grade
	Type VIII Collagenase	Sigma-Aldrich, Cat. No. C2139
	Dispase II	Roche, Mannheim, BW, Germany, Cat. No. 04942078001
	Trypsin inhibitor	Sigma-Aldrich, Cat. No. T6522
	DNase I	New England Biolabs, Ipswich, MA, USA, Cat. No. M0303S
EV Isolation and Purification	0.22 µm filter	Sartorius, Göttingen, Germany, Cat. No. 16541-K
	Polycarbonate centrifuge bottle	Beckman Coulter, Indianapolis, IN, USA, Cat. No. 355655
	Polypropylene microfuge tube	Beckman Coulter, Cat. No. 357448
	Paraformaldehyde (4% in PBS)	Sigma-Aldrich, Cat. No. P6148
Fixatives and TEM Reagents	Glutaraldehyde fixative (4%, EM Grade)	Solarbio, Beijing, China, Cat. No. P1127
	Uranyl acetate (2%)	Electron Microscopy Sciences, Hatfield, PA, USA, Cat. No. 22400
	Formvar/Carbon-coated Cu grids (200-mesh)	Electron Microscopy Sciences Cat. No. 71150
Antibodies for EV Characterization	RIPA buffer	Solarbio, Cat. No. R0010
	TSG101	Abcam, Cambridge, MA, USA, Cat. No. ab125011
	Calnexin	Abcam, Cat. No. ab22595
	β-actin	Cell Signaling Technology, Danvers, MA, USA, Cat. No. 4967
Protein and Western Blot Reagents	SDS–PAGE gels (10–12%)	Epizyme, Nanjing, Jiangsu, China, Cat. No. PG112
	PVDF membrane	Merck Millipore IPVH00010
	BCA Protein Assay Kit	Pierce, Thermo Fisher Scientific, Waltham, MA, USA, Cat. No. 23227
	ECL detection kit	Vazyme, Nanjing, Jiangsu, China, Cat. No. E411-04
	TBS-T	Solarbio, Cat. No. T1085-500 mL
	Low-binding microtubes (1.5 mL)	Eppendorf, Hamburg, Germany, Cat. No. 0030108116
Consumables and Plasticware	50/15 mL centrifuge tubes	Corning, Corning, NY, USA, Cat. No. 430828/430790
	100 µm cell strainer	Falcon, Corning, NY, USA, Cat. No. 352340
	Pasteur pipettes (3 mL)	Nest, Wuxi, Jiangsu, China, Cat. No. 318212
	Culture dishes (60 mm × 20 mm)	Corning, Cat. No. 430166
	Wide-bore pipette tips	Rainin, Mettler Toledo, Oakland, CA, USA, Cat. No. 30389218
	Trypsin (Sequencing grade)	Promega, Madison, WI, USA, Cat. No. V5111
Proteomics Reagents	C18 desalting columns	Thermo, Cat. No. 84850
	Formic acid (LC-MS grade)	Thermo, Cat. No. A117-50
	Acetonitrile (LC-MS grade)	Sigma-Aldrich, Cat. No. 1.00029.2500
Equipment	Ultracentrifuge	Beckman Coulter Optima XPN-100 Beckman Coulter Optima MAX-XP
	Refrigerated centrifuge (≤10,000× *g*)	Eppendorf 5910R
	Nanoparticle tracking analyzer (NTA)	Particle Metrix, Meerbusch, Germany, model: ZetaView
	Transmission electron microscope Zeta potential analyzer	Hitachi, Tokyo, Japan, model: HT7800 Malvern Panalytical, Malvern, UK, model: Zetasizer Pro
	Biosafety cabinet (Class II)	Thermo Fisher
	Constant-temperature shaker/incubator	Kylin-Bell, Haimen, Jiangsu, China
	Microplate Reader	BioTek, Winooski, VT, USA, model: SynergyLX
	UHPLC system (Easy-nLC 1200)	Thermo Fisher Scientific
	Orbitrap Exploris 480/Q extraction HF	Thermo Fisher Scientific

## Data Availability

The original data presented in the study are openly available in the ProteomeXchange Consortium via the iProX partner repository with the dataset identifier PXD079551.

## Supplementary Materials

The following supporting information can be downloaded at https://www.mdpi.com/article/10.3390/bioengineering13080879/s1, Figure S1. Proteomic profiling of mouse-derived EV and WCL fractions; Table S1. Human Raw Data; Table S2. Human Normalization; Table S3. Human Differentially Expressed Proteins; Table S4. Mouse Raw Data; Table S5. Mouse Normalization; Table S6. Mouse Differentially Expressed Proteins.

## Abbreviations

The following abbreviations are used in this manuscript:

EVs	Extracellular Vesicles (EVs)
PDAC	pancreatic ductal adenocarcinoma
TEM	Transmission Electron Microscopy
NTA	Nanoparticle tracking analysis
CV	Coefficient of variation
