# Supplementary material for: Isolation and Characterization of Extracellular Vesicles from Human and Mouse Pancreatic Tissue
Source: Bioengineering (Basel). 2026 Jul 30;13(8):879. doi: 10.3390/bioengineering13080879 (PMC13509241; doi:10.3390/bioengineering13080879)
Supplement: Supplementary file 1 [file bioengineering-13-00879-s001.zip › Figure S1.pdf]

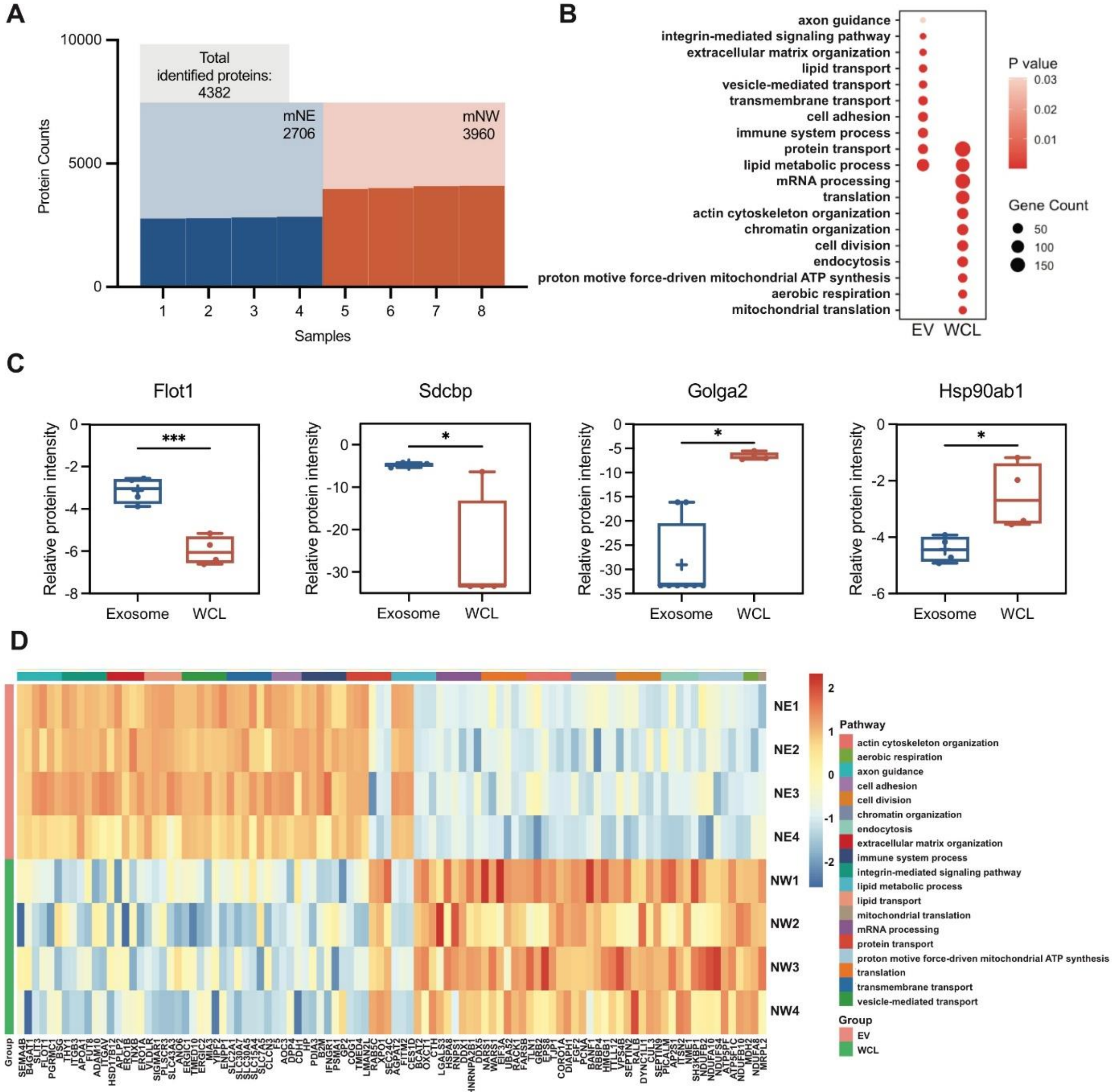

Figure S1. Proteomic profiling of mouse-derived EV and WCL fractions.

(A) Summary of the total number of differentially identified proteins across mouse EV （mNE) and WCL (mNW) samples.

(B) Gene Ontology biological process enrichment analysis of proteins enriched in mouse EV and WCL fractions. Dot size indicates gene count, and color represents enrichment P value.

(C) Relative protein intensity of EV-associated (FLOT1, SDCBP) and intracellular contaminant markers （GOLGA2, HSP90AB1) in mouse EV and WCL fractions.

(D) Heatmap showing representative proteins from highlighted biological pathways that were differentially expressed in both EV and WCL fractions.

Statistical significance was determined by a paired two-tailed Student’s t-test. *$P < 0.05$, ***$P < 0.001$.
